# A technique for preparing undecalcified osteochondral fresh frozen sections for elemental mapping and understanding disease etiology

**DOI:** 10.1007/s00418-022-02135-8

**Published:** 2022-07-09

**Authors:** Xiwei Fan, Kah Meng Lee, Michael W. M. Jones, Daryl Howard, Ross Crawford, Indira Prasadam

**Affiliations:** 1 Centre for Biomedical Technologies, School of Mechanical, Medical and Process Engineering, Queensland University of Technology, 60 Musk Ave/cnr. Blamey St, Kelvin Grove, Brisbane, QLD 4059 Australia; 2 Central Analytical Research Facility, Queensland University of Technology, Brisbane, 4059 Australia; 3 The Australian Synchrotron, Melbourne, 3168 Australia; 4 The Prince Charles Hospital, Brisbane, 4032 Australia

**Keywords:** Osteochondral interface, Trace elements, Zinc, Lead, Calcium, Strontium, Synchrotron

## Abstract

The anatomy of the osteochondral junction is complex because several tissue components exist as a unit, including uncalcified cartilage (with superficial, middle, and deep layers), calcified cartilage, and subchondral bone. Furthermore, it is difficult to study because this region is made up of a variety of cell types and extracellular matrix compositions. Using X-ray fluorescence microscopy, we present a protocol for simultaneous elemental detection on fresh frozen samples. We transferred the osteochondral sample using a tape-assisted system and successfully tested it in synchrotron X-ray fluorescence. This protocol elucidates the distinct distribution of elements at the human knee’s osteochondral junction, making it a useful tool for analyzing the co-distribution of various elements in both healthy and diseased states.

## Introduction

The osteochondral interface is becoming more widely recognized as a critical component involved in a variety of pathophysiologic events (Di Luca et al. 2015; Oliveira Silva et al. 2020). We previously demonstrated that distinct changes occurred with the progression of osteoarthritis (OA) (Fan et al. 2022). Advanced microimaging techniques are becoming increasingly important for assessing the pathophysiologic activity of tissues in detail. Synchrotron-based microimaging techniques are well suited for skeletal research owing to their ability to investigate the skeletal system with high throughput, high sensitivity, and accuracy (Ciani et al. 2018). Quantitative assessment of the microstructural properties of bone and cartilage in the osteochondral junction, such as geometry, and microstructural properties, such as relative elemental distribution, thickness, and connectivity, may improve our ability to estimate the quality of osteochondral regeneration (Wang et al. 2022; Fan et al. 2022).

Current protocols and studies describe various methods for scanning and analyzing resin-embedded osteochondral samples using confocal synchrotron radiation-induced micro X-ray fluorescence (SR-μXRF) (Roschger et al. 2013). Resin and paraffin are two traditional sample preparation methods for bone and cartilage (Vincic et al. 1989). However, these sample preparation methods entail complex chemical reactions that invariably alter the elemental composition (Hackett et al. 2011). As a result, the genuine changes in the elemental redistribution within the tissue are hard to determine. However, no study has focused on the elemental composition of the tissue owing to the difficulty of preparing fresh frozen osteochondral samples. The preparation of osteochondral tissue samples involves both soft and hard tissue. As a result, supporting material is always required; otherwise, the cartilage will always curl up to the bone part because of the tensile force of the two different tissues.

We developed a protocol in this article to provide a solution for observing the authentic changes of the osteochondral tissue. We provided a fresh frozen sample preparation solution for elemental detection and tested it with SR-μXRF, which provided fine images of multiple elements in both osteochondral tissues, proving to be a powerful tool for analyzing elemental changes in multiple osteochondral diseases.

## Materials and methods

**Fig. 1:**
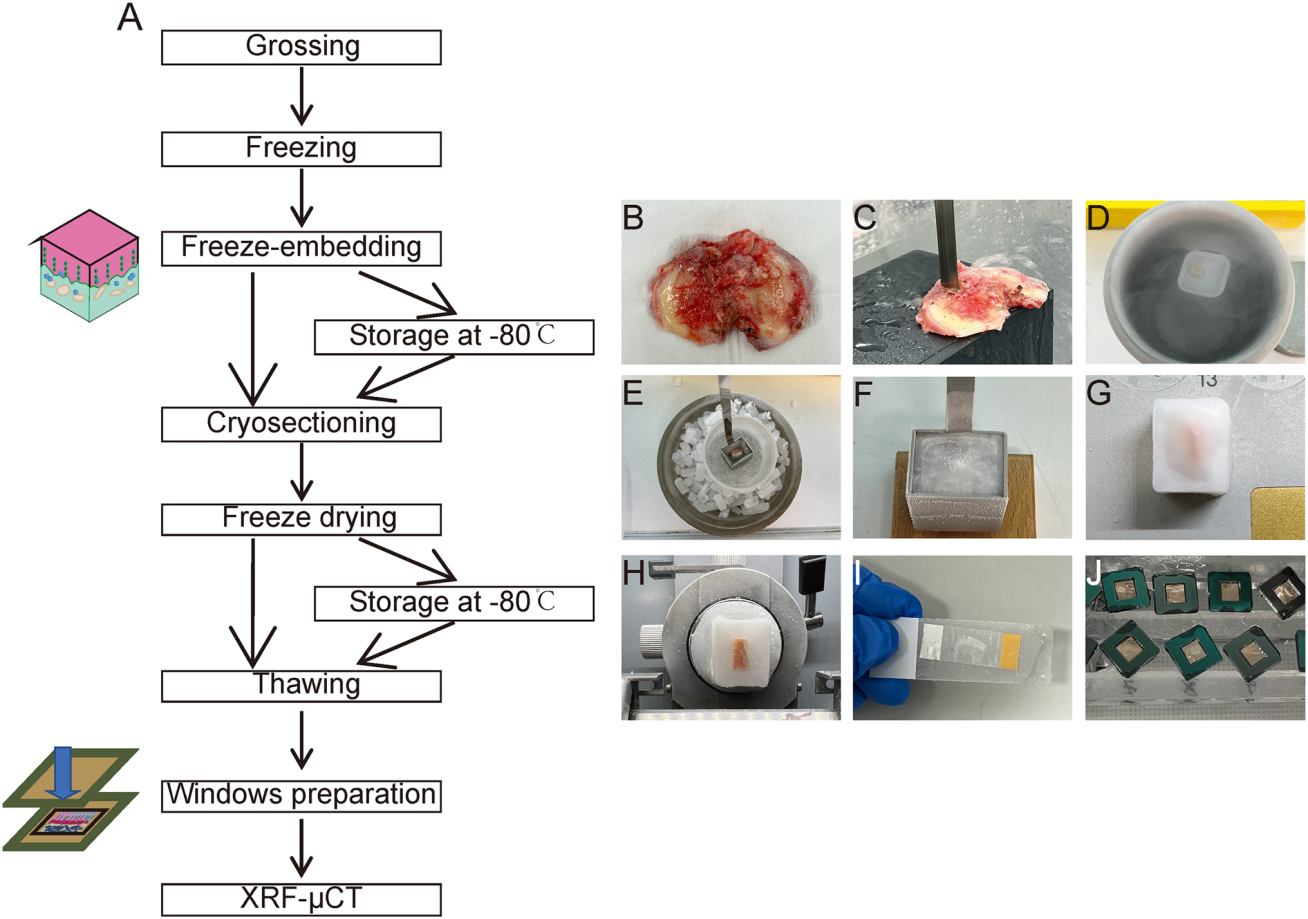
Sample preparation for the elemental mapping using the fresh frozen osteochondral unit. (**A**) Workflow for the sample preparation and synchrotron XRF examination. **(B)** Sample preparation using EXAKT bone saw. **(C)** Representative fresh frozen sample preparation methods for the osteochondral sample. **(D)** Representative freezing of the osteochondral sample using liquid nitrogen. **(E)** Representative freeze-embedding of the osteochondral sample. **(F)** Representative sample block of the osteochondral sample. **(G)** Representative sample preparation in cryostat. **(H)** Representative cryosectioning of the osteochondral unit. **(I)** Representative slides of the osteochondral sample after cryosectioning. **(J)** Representative sandwich structure for elemental mapping

After written approval was signed for the donation, a human lateral femoral condyle was obtained after total knee replacement from St Vincent Hospital (human ethics number #1,400,001,024). Nine patients were included in the study, all of whom underwent knee replacement due to OA. We graded the sample on the basis of the revised Mankin score. The samples were selected as G1 and G4 samples, in which G1 is the relatively intact knee joint with cartilage and subchondral bone, whereas G4 contains degraded cartilage and subchondral bone sclerosis mentioned in our previous publications (Fan et al. 2022). We used G1 samples with intact osteochondral samples for our protocol. None of the patients included in this study suffered from other inflammatory bone diseases. The flow chart is shown in Fig. 1A.

### Sample preparation

Immediately collect osteochondral sample containing bone and cartilage after surgery, and wash them with 1× phosphate-buffered saline (PBS) two times to rinse off excess blood on the surface (Fig. 1B).

Cut the sample into 1 cm × 1 cm × 1 cm cube using the EXAKT 310 Diamond Band Saw (EXAKT Apparatebau GmbH & Co.KG, Germany) (Fig. 1C).

Put the sample into ice-cold 1× PBS, and follow the steps below.

### Frozen tissue Sectioning

#### Sample freezing

- Label a storage plastic container at an approximate size of 2 cm × 2 cm × 2 cm. Add 4 ml of tap water into the container, and place the container into a −80 ℃ freezer for 3 h to freeze the water. This step is prepared in advance to maintain moisture in the chamber environment for long-term storage of frozen blocks.
- Prepare an esky of dry ice. The amount of dry ice is dependent on the number of samples to be frozen.
- Set up a fume hood with the blower turned on, and prepare the following chemicals and devices in the fume hood.
- Use a 200-ml plastic cylinder container to contain the hexane–dry ice mixture.
- Place the cylinder container on a metal bowl to contain any spillage from the hexane–dry ice mixture.
- Place the metal bowl with the cylinder container on a bluey underpad.
- Pour a sufficient amount of hexane into the cylinder container to cover the sample.
- Use tweezers to pick up dry ice pellets, and gently place them into hexane solution. The hexane solution will start to bubble upon the addition of dry ice.
- Use a digital thermometer to measure the temperature of the mixture. Once the temperature reaches approximately −76.8 ℃, begin the freezing process of the samples.
- On the basis of the Kawamoto protocol (Kawamoto and Kawamoto 2021), follow these steps:
- Place the sample into a mesh metal basket, and float the sample on a weigh boat on liquid nitrogen to be frozen for plunge freezing. Lift the frozen tissue up, and place it temporarily on top of the surface of dry ice in an esky (Fig. 1D).
- Pour a sufficient amount of super cryo-embedding medium (SCEM) (SECTION-LAB, Japan) into a metal mold of appropriate size.
- Gently place the metal mold with SCEM medium into hexane–dry ice mixture at two-thirds of the height of the metal mold until the boundary of SCEM is slightly frozen (Fig. 1E).
- Lift the metal mold up, place the frozen tissue into the SCEM in an appropriate position, and gently place the metal mold back into the hexane–dry ice mixture at two-thirds of the height of the metal mold until the SCEM is completely frozen.
- Lift the metal mold back up, and place it on a wood base with the base of the metal mold seated firmly against the knob of the wood base (Fig. 1F).
- Use a wood hammer to gently hammer on the metal mold to knock the frozen sample off the metal mold.
- Wipe the frozen sample with Kimwipes or a paper towel to eliminate excess hexane–dry ice mixture.
- Mark the region of interest (ROI) on the SCEM block, write label details on masking tape, wrap the block with aluminum foil, and tape the label around the foil.
- Repeat these steps for additional samples.

Take the storage container from a freezer, and place all frozen SCEM blocks in the container.

Store the container with samples in a −80 °C freezer for long-term preservation.

### Cryostat setup for sectioning

- Remove standard disposable blade holder from CryoStar NX70 (Thermo Scientific, USA) using an Allen key.
- Replace with a knife holder, place it at an angle at 12.5°, and tighten with an Allen key. This angle has been optimized and tested to provide smooth sectioning of calcified samples without resistance.
- Slide a tungsten carbide knife, D profile (Dorn & Hart Microedge, USA) into the knife holder, and tighten the knobs on the sides to secure the knife. Ensure that the blade guard is up to prevent injury.
- Adjust the cryostat temperature settings to the following:
- Specimen temperature: −30 °C
- Knife temperature: −28 °C. Leave the temperature adjustment for 30 min

While the cryostat temperatures are adjusting, remove a frozen embedded SCEM sample from a storage container and place a few drops of SCEM onto the center of a chuck (Fig. 1G).

Attach the frozen block’s non-region of interest (ROI) side to the chuck and place it on the cryobar. Tap the cryobar icon on the cryostat’s control panel to start the frozen block’s quick freezing to chuck.

Insert the chuck with frozen block onto the specimen head holder of the sectioning head, and bring the lever down to clamp the chuck tightly to the specimen head holder. Leave the chuck with the frozen block on the specimen holder for 20–30 min to habituate to the temperature of the specimen head on the cryostat.

### Cryosectioning

- Use the joystick on the cryostat to move the tungsten carbide knife close to the frozen block, and adjust the frozen block so that it sits flushed against the knife.
- Select trim mode, adjust trim thickness to 20–30 μm, and trim until ROI is exposed.
- Once ROI is exposed, switch to section mode and adjust section thickness to 10 μm (Fig. 1H).
- Sacrifice the first section after thickness has just been adjusted as the thickness of the first section is normally not reflective of the selected thickness.
- Following the Kawamoto technique (Kawamoto and Kawamoto 2021):
- Cut an appropriate size of Kawamoto’s cryofilm tape [3C(16UF)] that fits the size of the sample by ensuring there is enough space surrounding the tissue.
- Peel off the nonsticky plastic sheet from the cryofilm tape.
- Hold onto the nonsticky part of the tape, and gently bring it close to the surface of the frozen block. The sticky part of the tape is strongly attracted to the surface of the SCEM block.
- Use the deerskin roller to gently roll on the tape glued to the surface of the block. Ensure that the rolling is done in the same direction. For example, if it is done from top to bottom, then perform this direction repeatedly. Do not perform from top to bottom and then from bottom to top direction, as this can accidentally scrunch up the tape glued to the block surface. The sample section will not stay intact on the tape if that happens (Fig. 1I).
- Once the tape adheres properly to the block surface, gently section the block and ensure that the nonsticky part of the tape does not get caught on the knife.
- Place the tape with the section adhered in the cryostat to keep the temperature of the section cool at −20 °C.

### Storage of sections

Attach the tape (non-section side) onto a slide using a glue stick (Marbig, China), and store these slides in a secured slide box. Place the slide box into a −80 ℃ freezer for extended storage.

### Synchrotron sample preparation

Place the slides in a 50 ml Falcon tube. Cover the falcon tube with Parafilm. Drill several holes on the film with a needle to preserve the slides from the vacuum, and freeze-dry the sample gently.

Place the Falcon tube into the freeze-dryer. Adjust the pressure to 0.1 mPa. Freeze-dry the sample using the freeze-dryer overnight.

**Tip**: Do not expose the sample at room temperature for too long since it may attract water behind the tape, thus interfering with the experimental results.

Place the sample at room temperature. Use the scalpel blade to cut the tape to fit the size of the Si_3_N_4_ window.

Cover the Si_3_N_4_ windows (5.5 mm, 200 nm 10 μm thickness, 200 mm frame thickness; Australian National Fabrication Facility, QLD, Australia) to have the sample attach to the window properly. The windows can be stored at room temperature. Then place the window on the frame designed for the synchrotron. Remember to align the long axis of the sample with the orientation of the scanning (Fig. 1J).

Samples are ready to analyze.

### X-ray fluorescence microscopy data collection

XRF photons were collected as an event mode data stream at the SR-μXRF beamline of Australian Synchrotron (Howard et al. 2020) using the Maia detector system (Ryan et al. 2018) and processed using the dynamic analysis method (Ryan and Jamieson 1993) as implemented in GeoPIXE (Ryan et al. 2005). The data were quantified using well-characterized metallic foils and exported as 32-bit tiffs with areal density units of ng cm^−2^.

### X-ray fluorescence microscopy image cleaning

XRF photons were collected as an event mode data stream at the XFM beamline of Australian Synchrotron (Howard et al. 2020) using the Maia detector system (Ryan et al. 2018) and processed using the dynamic analysis method (Ryan and Jamieson 1993) as implemented in GeoPIXE (Ryan et al. 2005). The data were quantified using well-characterized metallic foils and exported as 32-bit tiffs with areal density units of ng cm^−2^. The XFM beamline operates in the atmosphere with a low energy detection limit of approximately 1.5 keV, limiting detection to elements heavier than silicon.

## Results and discussion

Our method can effectively reveal the distribution of various types of elements in fresh frozen osteochondral tissue of the human knee joint. The elemental distribution patterns in the samples are distinct without alteration through chemical fixation (Ref Hackett et al., Analyst 2011 as above).

### Co-distribution of multiple elements in the normal osteochondral interface

**Fig. 2:**
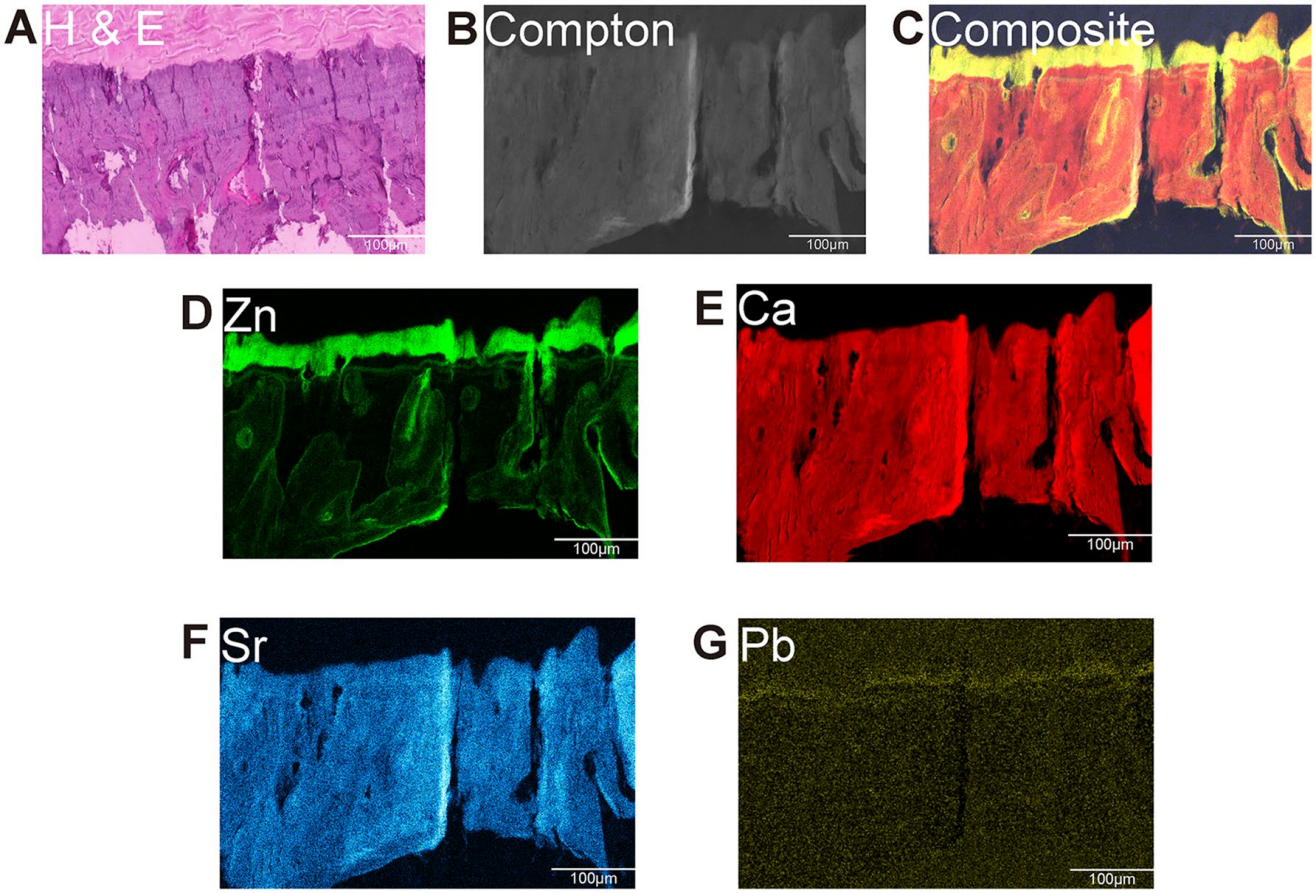
Elemental mapping for the osteochondral unit in the normal group**. (A)** Representative H&E staining of the osteochondral unit (*n* = 9). **(B)** Representative elemental mapping images of Compton osteochondral unit (*n* = 9). **(C)** Representative elemental mapping merged images of osteochondral unit (*n* = 9; Zn, Ca, Sr, Pb). **(D)** Representative elemental mapping images of Zn osteochondral unit (*n* = 9). **(E)** Representative elemental mapping images of Ca osteochondral unit (*n* = 9). **(F)** Representative elemental mapping images of Sr osteochondral unit (*n *= 9). **(G)** Representative elemental mapping images of Pb osteochondral unit (*n* = 9). Scale bar, 100 μm

Our protocol was verified to preserve all related elements in the osteochondral interface. We also performed hematoxylin and eosin (H&E) staining for the reference (Fig. 2A). All elements displayed high signal-to-noise ratio images, particularly for abundant elements such as calcium, phosphorus, strontium, zinc, and others (Fig. 2B). We discovered a distinct pattern of zinc, calcium, strontium, and lead (Fig. 2C). This is the first description of elemental distribution in high-resolution elemental imaging of the osteochondral interface using a fresh frozen sample.

Our findings demonstrate that zinc is distributed in a specific pattern at the osteochondral interface. We discovered a very high abundance in the tidemark, a low abundance in calcified cartilage, and a very high abundance in the subchondral bone. Zinc distribution was uniform throughout the osteochondral interface Fig. 2D). Zinc accumulates in the tidemark area, cartilage cavity in the calcified cartilage zone, and subchondral bone area, whereas no zinc is accumulated in the matrix of the calcified cartilage zone. Additionally, we observed a high concentration of zinc in the cement line region, which is consistent with the study of Pemmer et al. (Pemmer et al. 2013). They observed a similar spatial distribution in the cement line of the Haversian system.

Additionally, we discovered an accumulation of zinc in the chondrocyte and bone cavities, which contain chondrocytes and osteocytes. Calcium was evenly distributed throughout the calcified cartilage layer and subchondral bone (Fig. 2E). There was no discernible difference between the two regions. Additionally, the calcium elemental mapping lacks a distinct contour between calcified cartilage and subchondral bone. We observed similar distribution of strontium in the osteochondral junction (Fig. 2F).We observed an accumulation of trace element lead in the deep layer of tidemark (Fig. 2G), which is consistent with the findings of Roschger et al. (Roschger et al. 2013).

### Quantifying the elemental mapping allows for more precise analysis in osteochondral experiments.

**Fig. 3:**
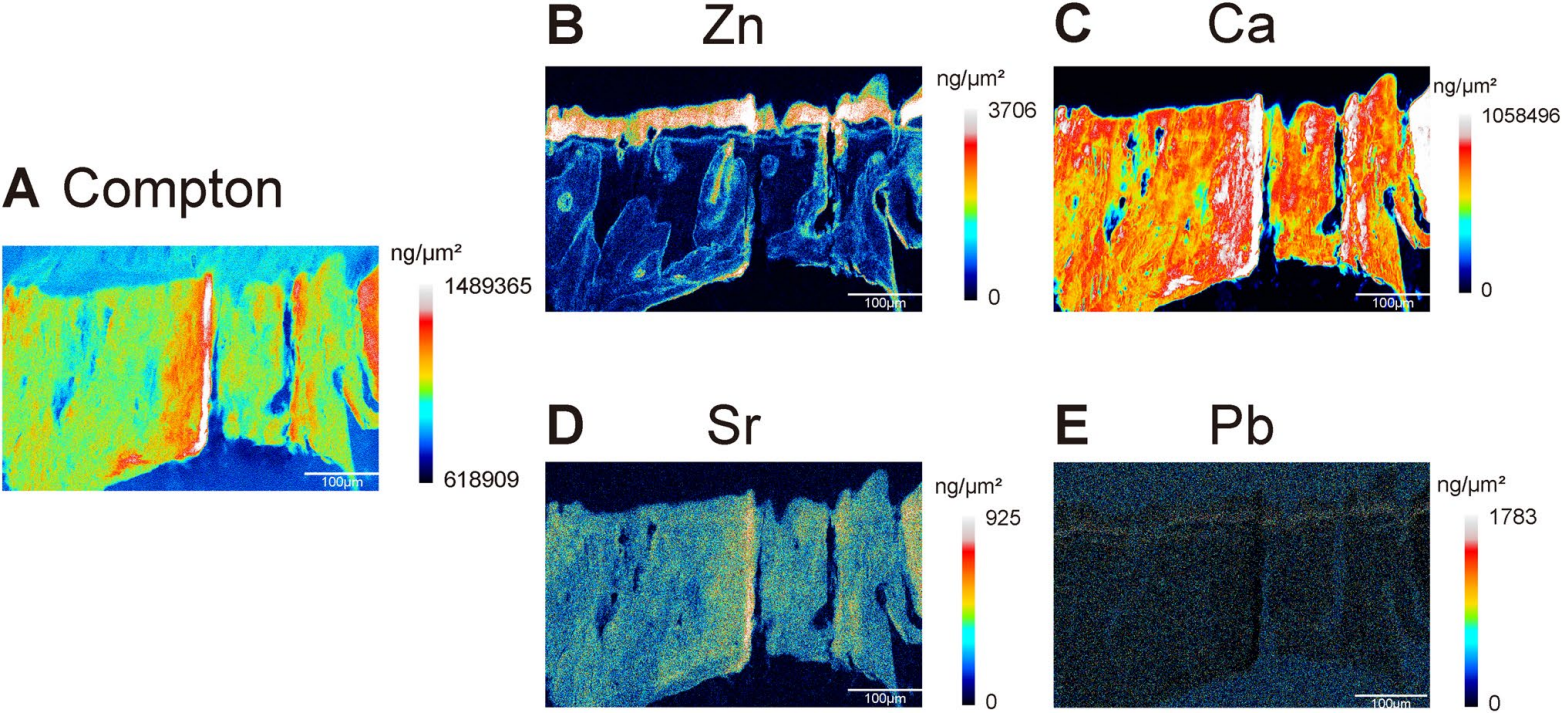
Quantification for elemental mapping in the osteochondral unit in the normal osteochondral unit. **(A)** Quantitative Compton elemental mapping images of normal osteochondral unit (*n* = 9). **(B)** Quantitative Zn elemental mapping images of normal osteochondral unit (*n* = 9). **(C)** Quantitative Ca elemental mapping images of normal osteochondral unit (*n *= 9). **(D)** Quantitative Sr elemental mapping images of normal osteochondral unit (*n* = 9). **(E)** Quantitative Pb elemental mapping images of normal osteochondral unit (*n* = 9). Scale bar, 100 μm

Quantified elemental distributions allow for additional analysis of the fine area (Fig. 3A). This includes quantitative analysis of specific areas and segmentation of regions based on their differences. We get a distinct distribution pattern of zinc using quantification imaging. Zinc abundance ranges from 0 to 3706 ng/μm^2^ in the osteochondral interface (Fig. 3B), calcium abundance ranges from 0 to 1,058,496 ng/μm^2^ (Fig. 3C), strontium abundance ranges from 0 to 925 ng/cm^2^ (Fig. 3D), and lead abundance ranges from 0 to 1783 ng/cm^2^ (Fig. 3E).

Specific critical points can determine whether or not an experiment succeeds. Water is a significant contaminant that can lead to radiation-induced damage (Jones et al. 2017). As a result, the samples must be freeze-dried prior to sectioning and mounted at room temperature on the windows. This is a critical step in avoiding water condensation as a result of the low temperature. The images shown here were taken in the atmosphere. However, the data can be collected in the vacuum under some circumstances, resulting in images with a higher signal-to-noise ratio.

Another critical point was the procedure for sample preparation. We used a tape-assisted system developed by Kawamoto et al. (Kawamoto and Kawamoto 2021) to assist with the sample transfer. The tape and sample combination was transferred to the silicon nitride window with the tape positioned behind the sample. A second window was placed on top of the sample to secure the sample position. After that, the two windows were secured together. This arrangement ensured that the fluorescent photons had to pass only through a single window and did not have to pass through the tape before being detected. It was the first time the system was used for synchrotron-based elemental mapping, demonstrating the reliability of the system.

This paper has some limitations. Chemical fixation has been shown to alter the endogenous element distributions in soft tissue (Hackett et al. 2011), and while a similar study does not exist for calcified tissue, we still anticipate that there would be elemental alterations in line with soft tissue, especially in the cartilage. While we cannot quantify the benefit for calcified tissue at this stage, we believe that the possibility that resin embedding results in a change in the endogenous elemental distributions are not ignorable. Therefore, the current paper provides a method of investigating the osteochondral interface using fresh frozen osteochondral samples without any compromise to the sample preparation.

To summarize, we developed an elemental mapping protocol for fresh frozen tissue that preserves the natural characteristics and demonstrated suitability for subsequent identification and analysis using osteochondral tissue. This protocol is applicable to all calcified tissue in humans and rats, providing new insight into the etiology and mechanism of the disease.

## Data Availability

All essential data are included in the manuscript. On reasonable request, any remaining information can be acquired from the corresponding author.
